# The Prevalence of Insulin Resistance in Malaysia and Indonesia: An Updated Systematic Review and Meta-Analysis

**DOI:** 10.3390/medicina58060826

**Published:** 2022-06-19

**Authors:** Lucky Poh Wah Goh, Suraya Abdul Sani, Mohd Khalizan Sabullah, Jualang Azlan Gansau

**Affiliations:** Faculty of Science and Natural Resources, University Malaysia Sabah, UMS Road, Kota Kinabalu 88400, Malaysia; luckygoh@ums.edu.my (L.P.W.G.); suraya.abdulsani@ums.edu.my (S.A.S.); khalizan@ums.edu.my (M.K.S.)

**Keywords:** insulin resistance, meta-analysis, prevalence, Southeast Asia

## Abstract

*Background and Objectives:* Noncommunicable diseases such as diabetes are strongly associated with the insulin resistance (IR) status of an individual. However, the prevalence of insulin resistance in Southeast Asia is poorly reported. Hence, this study investigated the prevalence of IR in Southeast Asia from the year 2016 to 2021. *Materials and Methods:* This study was carried out according to PRISMA guidelines. The literature search was conducted utilizing the PubMed and SCOPUS databases from the year 2016 to 2021 using the keywords ‘(insulin AND resistance) OR (insulin AND sensitivity) OR (prevalence OR incidence) AND (Malaysia OR Thailand OR Singapore OR Brunei OR Cambodia OR Indonesia OR Laos OR Myanmar OR Philippines OR Timor leste OR Vietnam)’. Funnel plot and publication bias were assessed using Egger’s tests. Data were expressed as the prevalence rate. *Results:* A total of 12 studies with 2198 subjects were considered in the meta-analysis. Significant heterogeneity (I^2^ > 94% and *p*-value < 0.001) was observed in the meta-analysis. The overall prevalence of IR in Southeast Asia was 44.3%, with Malaysia having the highest prevalence rate at 50.4%, followed by Indonesia at 44.2%. Bias was detected in the meta-analysis. It may be that reports published before the year 2016 met the study selection criteria, but were excluded from the meta-analysis. *Conclusions:* The results from the meta-analysis indicate that the prevalence of IR in Southeast Asia is very high. This provided insights for healthcare policy makers and public health officials in designing IR screening programs.

## 1. Introduction

Noncommunicable diseases (NCDs) are expected to proportionally increase with the increasing number of people aged 60 years and above in Asia [1]. The aging population is expected to double by 2050, at which point it would encompass one-third of the total human population [2]. Aging populations lead to the increased prevalence of NCDs, which have a serious impact on the public health system and national productivity. NCDs are chronic diseases that develop over time. The main types of NCDs with a high annual mortality rate worldwide are cardiovascular diseases (17.9 million), cancer (9.3 million), respiratory diseases (4.1 million), and diabetes (1.5 million) [3].

Diabetes is defined as an uncontrollable level of glucose in the bloodstream due to inadequate insulin production by the pancreas [4]. There are mainly two major types of diabetes, type 1 (destruction of insulin-producing cells in the pancreas) and type 2 (combination of insulin resistance (IR) and insufficient insulin production). IR is defined as an impaired response towards insulin stimulation by the target tissues and could cause many diseases including diabetes. Type 2 diabetes (T2D) is the most common form of diabetes that occurs in older adults. Globally, the prevalence rate of T2D was 6059 cases per 100,000 individuals in 2017 and is projected to increase to 7079 cases per 100,000 individuals by 2030 [4]. The prevalence of T2D has been shown to be correlated with the socioeconomic development of a region, where developed regions such as Western Europe have a high IR prevalence rate compared to developing regions such as Southeast Asia [4]. However, increasing economic growth in developing Southeast Asian countries such as Indonesia, Malaysia, Thailand, and Vietnam has been observed with an increased T2D prevalence rate [4]. This indicates that public health measures for the management and treatment of T2D are required for developing Southeast Asian countries in the future.

T2D patients can be treated through changes in lifestyle, and prescriptions of oral or injectable medications [5]. It is worth noting that although IR does not necessarily cause T2D, metformin, a drug commonly used in diabetes treatment, does not improve insulin sensitivity and renders the treatment for IR ineffective [6,7,8]. The worldwide prevalence of IR ranges from 15.5% to 46.5% [9,10,11]. IR has also been associated with all-cause mortality at a rate of 20.6% to 25.3% [12]. IR is a highly variable medical condition, which is due to multiple factors, such as physical activity, stress, and sleep sufficiency [13]. Therefore, understanding the prevalence of IR has a significant impact on public health policy.

The prevalence of IR has been poorly explored and reported in the Southeast Asia region. Developing nations located in the Southeast Asia region have been shown to have an increased rate of T2D, which could lead to IR-associated deaths in the future. This poses a significant public health risk regarding the treatment and management of IR in the future. Hence, this study investigated the prevalence of IR among Southeast Asian countries using a systematic meta-analysis method with adherence to preferred reporting items for systematic review and meta-analysis (PRISMA) guidelines.

## 2. Materials and Methods

### 2.1. Guidelines and Study Search

The study was conducted, and the results are reported according to PRISMA guidelines [14]. The literature search was conducted utilizing the PubMed and SCOPUS databases. The time period was limited to 1 January 2016 until 1 June 2021. The keywords used in the literature search for both PubMed and SCOPUS databases were (insulin AND resistance) OR (insulin AND sensitivity) OR (prevalence OR incidence) AND (Malaysia OR Thailand OR Singapore OR Brunei OR Cambodia OR Indonesia OR Laos OR Myanmar OR Philippines OR Timor leste OR Vietnam).

### 2.2. Study Filtering and Extraction of Data

**Figure 1:**
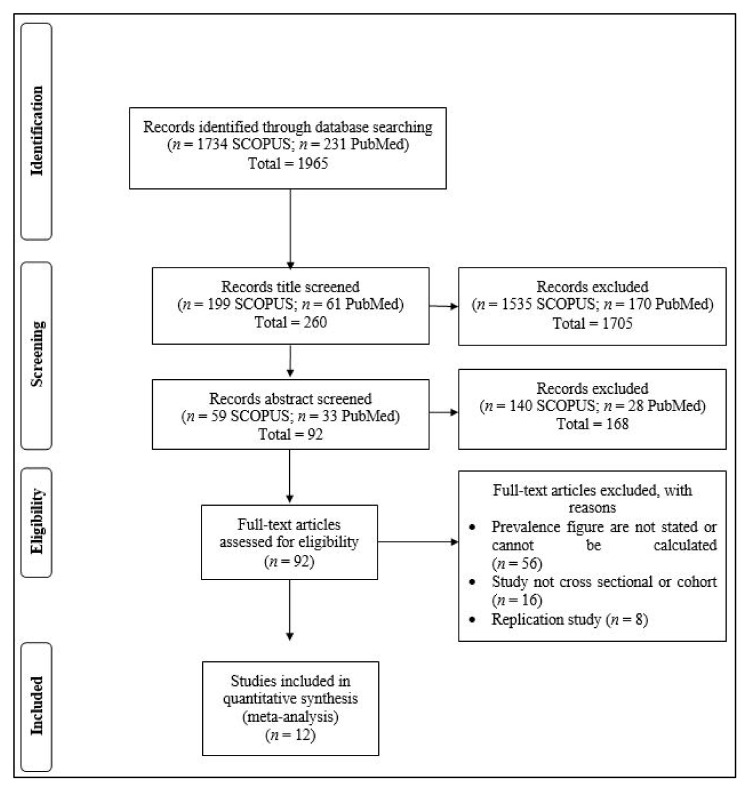
PRISMA flow diagram of systemic literature search from 1 January 2016 to 1 June 2021.

Two investigators screened the literature search results and further reviewed potential studies. All literature was independently screened by the authors, and any inconsistent reviewed findings were further scrutinized. Firstly, all the titles of the literature were initially screened, and literature that fulfilled the criteria was subjected to the following abstract screening process. Full texts which were eligible to be included in the study were further screened, and only studies that fulfilled the criteria were taken into account in this study. The inclusion criteria were as follows: (1) literature published in the English language; (2) peer-reviewed publications only; (3) the prevalence of IR or the prevalence rate of IR can be calculated from the data presented in the article; and (4) all age groups and all populations within the searched Southeast Asian countries were included. The exclusion criteria were: (1) non-cross-sectional, observational, or cohort article. The author name, year of publication, country, total sample size, method used in determining IR, and number of IR individuals were extracted and recorded. The literature filtering and review processes are illustrated in Figure 1.

### 2.3. Data and Statistical Analysis

Prevalence of IR was calculated for studies included in the meta-analysis. The I^2^ index (%) and Q test (*p*-value) were calculated to determine the heterogeneity between the studies. The random-effects model was used for calculating the prevalence of IR if high heterogeneity was observed as determined by an I^2^ value of more than 75% and Q test (*p*-value < 0.1) [15]. The prevalence of IR in each study is illustrated using a forest plot, with a 95% confidence interval. The publication bias was investigated by employing a funnel plot and Egger’s tests of asymmetry [16]. Comprehensive Meta-Analysis version 2 software was used for all analyses undertaken [17].

## 3. Results

### 3.1. Characteristics of Studies Included

A total of 12 studies from 1 January 2016 to 1 June 2021, comprising 2198 subjects, were included in this meta-analysis. The literature search followed by the review process yielded studies from Indonesia and Malaysia. Studies from other Southeast Asian countries were not present because they did not fulfill the selection criteria. The characteristics of included studies are shown in Table 1. All of the studies utilized the homeostatic model assessment for insulin resistance (HOMA-IR) for evaluation of IR in the subjects. Rahmadhani et al., 2017 was the largest study with a total number of 795 subjects; however, it had a low prevalence rate of 21.0% [17].

### 3.2. Study Heterogeneity and Prevalence of IR

**Figure 2:**
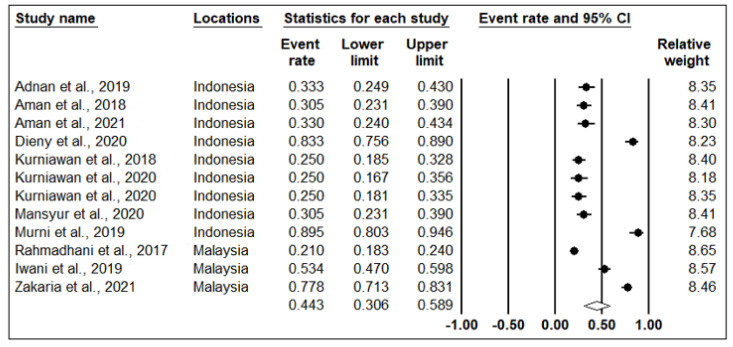
Forest plot of overall insulin resistance prevalence using random-effects model. The filled circle represents the prevalence rate of IR in each study. Unfilled diamond is the combined prevalence rate of IR [18,19,20,21,22,23,24,25,26,27,28,29].

**Figure 3:**
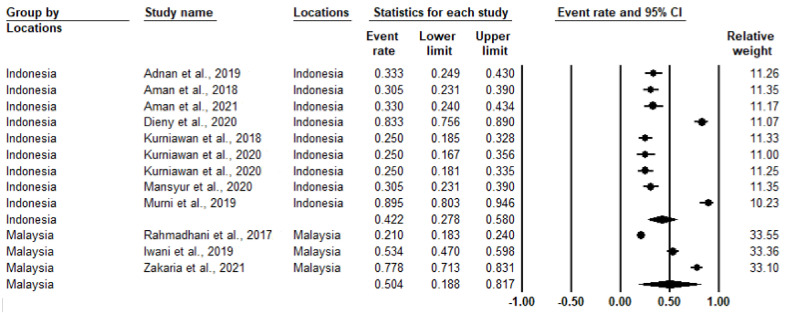
The insulin resistance prevalence grouped according to country using random-effects model as illustrated by the forest plot. The filled round circle is the prevalence of IR with 95% CI. The filled diamond represents the overall prevalence for subgroups [18,19,20,21,22,23,24,25,26,27,28,29].

Significant heterogeneity was observed in the meta-analysis, with I^2^ > 94% and *p*-value < 0.001 (Table 2). Therefore, the random-effects model was used to conduct the meta-analysis. The overall prevalence of IR was 44.3% (I^2^ = 97%; *p*-value < 0.001) (Figure 2). Subgroup analysis based on specific countries shows that Malaysia had the highest prevalence of IR at 50.4%, followed by Indonefsia, with an IR prevalence of 42.2% (Figure 3). Although Rahmadhani et al. (2017) had the largest number of samples, they had a similar relative weight in both Figure 2 and Figure 3 [17].

### 3.3. Publication Bias

**Figure 4:**
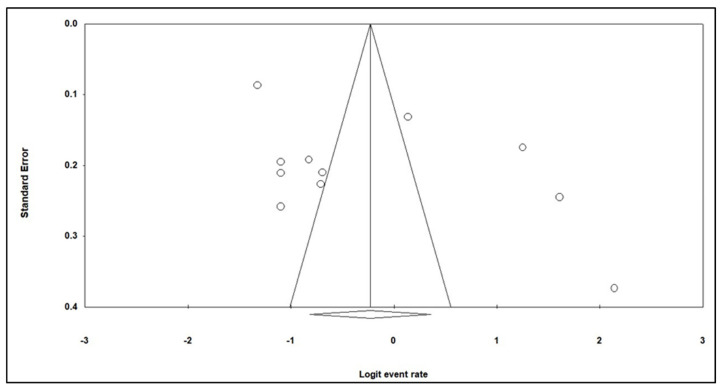
Funnel plot of standard error by logit event rate of this study. The unfilled dots represent the studies included.

Egger’s tests and a funnel plot were used to estimate any publication bias of the included studies in this meta-analysis. The shape of the funnel plot did not illustrate symmetry, suggesting there was a potential for publication bias (Figure 4). This was further supported by Egger’s tests, with a *t*-value = 1.70 and a *p*-value = 0.12, which indicated potential publication bias existed in this meta-analysis.

## 4. Discussion

There remains a lack of studies and reports on the prevalence of IR in the Southeast Asian region, since most countries in Southeast Asia are considered developing nations. IR is caused by dietary and hormone changes, as well as metabolic diseases such as diabetes [30]. The consequence of IR can lead to the development of T2D, and IR precedes the development of T2D by 10 to 15 years, which could be due to the failure of β cells [31]. There are multiple mechanisms that cause IR, such as suppression of lipolysis, cellular uptake of available plasma glucose, and net glucose synthesis [32]. This leads to increased insulin secretion by β cells in the pancreas. However, the consistent ongoing loop of elevated insulin production by β cells and IR eventually causes β-cell failure due to toxicity, leading to overt T2D [33].

IR exerts a heavy economic burden on a nation. For the years 2013 to 2014, expenditure and hospitalization rates were three times higher for IR patients compared to those for non-IR patients, regardless of the type of morbidity [34]. Analysis from the years 2012 to 2017 revealed that the economic costs of diabetes increased by 26% [35]. Furthermore, financial burdens are even greater when considering the intangible costs of IR cases, such as the pain and suffering, self-paid caregiver resources, and undocumented cases.

The prevalence of T2D is tied to socioeconomic development status across the world. Developed nations in Western Europe have a higher T2D prevalence when compared with developing regions in Southeast Asia [4]. However, increased socioeconomic status of Southeast Asian countries such as Indonesia, Malaysia, Thailand, and Vietnam was observed with increasing prevalence of T2D [4].

The prevalence of IR in a population is poorly explored as compared to T2D. However, IR is one of the main risk factors that leads to the development of T2D. Although drugs such as metformin could increase insulin sensitivity, contrasting reports on the ineffectiveness of increasing insulin sensitivity have been emerging [8]. This indicates that public health measures for the management and treatment of T2D and IR will be challenging in the future.

The symmetrical shape of the funnel plot indicates that minimal bias exists in this meta-analysis. This was further supported by Egger’s test (*t*-value = 1.70 and *p*-value = 0.12), where no significant bias exists. Meta-analysis bias could skew the findings of a study, leading to unreliable data. However, this meta-analysis did not present a significant bias that skews the data analyzed. The random-effects model was used in the meta-analysis due to the significant heterogeneity observed. The model assumes that true effects could vary between studies [36].

The meta-analysis observed that the prevalence rate of IR was 44.3% in the Southeast Asian region, which is considered significantly high given that the worldwide IR prevalence rate ranges from 15.5 to 46.5% [9,10,11]. Further meta-analysis stratified according to country observed that the prevalence rate of IR in Malaysia was 50.4%. This was higher than the worldwide prevalence rate, whereas the neighboring country Indonesia had a lower prevalence rate of 42.4%. The high prevalence rate of IR is concerning, as it is one of the main risk factors for developing T2D, which causes a great economic burden [4]. The levels of IR are governed by various factors, such as exercise, fasting, and emotional stresses [13].

Other than diabetes, IR is also associated with other diseases, such as metabolic syndrome, hypertension, atherosclerosis, and cardiovascular diseases [37]. IR patients were reported to have a higher risk of developing cardiovascular diseases and increase spending on hospitalization and health care bills by three times when compared to insulin-sensitive patients [34]. The economic burden and impact of IR has been reported to be significantly higher than the impact of metabolic syndrome and cardiovascular disease [34]. Metabolic syndrome is complex, and the etiology remains unclear, but it is defined as clusters of risk factors such as obesity, hyperinsulinemia, hypertension, and dyslipidemia. However, IR is a known common mechanism which plays a central role in these syndromes [38]. Hence, this systematic review has revealed the importance of IR and the lack of reports, which warrants the attention of the public health official in designing IR screening programs.

IR is a complex condition with multiple onset mechanisms and could potentially lead to the development of other diseases. Therefore, generating information on the prevalence of IR would yield enormous benefits to a nation and society engaging in efforts towards designing healthcare policies, managing diseases related to IR, and preventing IR, so the risk of developing other diseases can be greatly reduced. There are many methods of evaluating IR; HOMA has proven to be a great clinical tool and become the standard for the assessment of the IR status of an individual [39]. However, the interpretation of HOMA-IR should be performed carefully by clinicians as individuals may have underlying diseases such as pancreatic dysfunction or be receiving prescribed drugs [39].

There are strengths and limitations of this meta-analysis. Firstly, the stringent criteria for including studies in this meta-analysis only included studies from Malaysia and Indonesia. Studies reporting IR prevalence from other countries did not fulfill the criteria; hence, the prevalence rate of the IR calculated in this study was localized to the Southeast Asia region as well as Malaysia and Indonesia. These stringent criteria ensure that quality data were obtained and concurrently highlighted that the prevalence of IR in Southeast Asia is poorly reported because limited studies were found in the literature. However, only studies from the year 2016 to 2021 and SCOPUS and PubMed databases were screened in this meta-analysis study. Therefore, it remains possible that reports published before the year 2016 as well as other databases met the study selection criteria; however, these were excluded in the meta-analysis. This is due to the evidence that prevalence of IR or diabetes is strongly associated with the socioeconomic status of a country, and economic growth progresses over time. Hence, this meta-analysis focused on investigating the prevalence of IR in the last five years, because including studies prior to 2016 might have obscured the prevalence of IR in a region. We did not stratify the prevalence of IR according to other pathological diseases or perform study quality and bias filtering due to the limited reports available.

## 5. Conclusions

The present study reported the prevalence of IR in Southeast Asia using a systematic review and meta-analysis approach, and it was found that a high prevalence of IR requires attention from health authorities due to the potential onset of other diseases caused by IR as well as the economic burden of IR. This meta-analysis also highlighted that studies reporting the prevalence of IR in Southeast Asia are limited, as demonstrated by the inclusion of only two Southeast Asian countries in the meta-analysis. We emphasize that further investigation of the prevalence of IR in other countries should be strongly considered in future work. The findings of this meta-analysis are beneficial to governmental and non-governmental bodies in designing research programs for IR.

## Figures and Tables

**Table 1:** The references and main attributes of included studies.

Study (References)	Method of Defining Insulin Resistance	Country	Events ^1^	Total^ 2^
Adnan et al., 2019 [18]	HOMA-IR	Indonesia	34	102
Aman et al., 2018 [19]	HOMA-IR	Indonesia	39	128
Aman et al., 2021 [20]	HOMA-IR	Indonesia	29	88
Dieny et al., 2020 [21]	HOMA-IR	Indonesia	100	120
Kurniawan et al. 2018 [22]	HOMA-IR	Indonesia	35	140
Kurniawan et al., 2020a [23]	HOMA-IR	Indonesia	20	80
Kurniawan et al., 2020b [24]	HOMA-IR	Indonesia	30	120
Mansyur et al., 2020 [25]	HOMA-IR	Indonesia	39	128
Murni et al., 2019 [26]	HOMA-IR	Indonesia	68	76
Rahmadhani et al., 2017 [27]	HOMA-IR	Malaysia	167	795
Iwani et al., 2019 [28]	HOMA-IR	Malaysia	124	232
Zakaria et al., 2021 [29]	HOMA-IR	Malaysia	147	189
		Total	832	2198

^1^ Number of insulin-resistant individuals. ^2^ Total number of subjects in the study.

**Table 2:** The heterogeneity and prevalence rate of insulin resistance in the overall and subgroups of the meta-analysis.

Heterogeneity		Prevalence Rate (95% CI)	Sample Size (*n*)	Number of Studies	Subgroups
I^2^ (%)	*p*-Value				
97.0	<0.001	0.443 (0.306–0.589)	2198	12	Overall
94.80	<0.001	0.422 (0.278–0.580)	982	9	Indonesia
99.06	<0.001	0.504 (0.188–0.817)	1216	3	Malaysia

## Data Availability

Not applicable.
